# Unraveling the specialized metabolic pathways in medicinal plant genomes: a review

**DOI:** 10.3389/fpls.2024.1459533

**Published:** 2024-12-24

**Authors:** Mingcheng Wang, Shuqiao Zhang, Rui Li, Qi Zhao

**Affiliations:** ^1^ Institute for Advanced Study, Chengdu University, Chengdu, China; ^2^ Engineering Research Center of Sichuan-Tibet Traditional Medicinal Plant, Chengdu University, Chengdu, China; ^3^ School of Food and Biological Engineering, Chengdu University, Chengdu, China

**Keywords:** medicinal plants, specialized metabolites, multi-omics integration, biosynthesis pathways, high-throughput genome sequencing

## Abstract

Medicinal plants are important sources of bioactive specialized metabolites with significant therapeutic potential. Advances in multi-omics have accelerated the understanding of specialized metabolite biosynthesis and regulation. Genomics, transcriptomics, proteomics, and metabolomics have each contributed new insights into biosynthetic gene clusters (BGCs), metabolic pathways, and stress responses. However, single-omics approaches often fail to fully address these complex processes. Integrated multi-omics provides a holistic perspective on key regulatory networks. High-throughput sequencing and emerging technologies like single-cell and spatial omics have deepened our understanding of cell-specific and spatially resolved biosynthetic dynamics. Despite these advancements, challenges remain in managing large datasets, standardizing protocols, accounting for the dynamic nature of specialized metabolism, and effectively applying synthetic biology for sustainable specialized metabolite production. This review highlights recent progress in omics-based research on medicinal plants, discusses available bioinformatics tools, and explores future research trends aimed at leveraging integrated multi-omics to improve the medicinal quality and sustainable utilization of plant resources.

## 1 Introduction

Plants have inhabited Earth significantly longer than humans, with a staggering diversity of over 300,000 plant species spread across various ecosystems. Throughout the history of human civilization, a deep connection has formed between plants and humans. Humans rely on the oxygen produced by plants through photosynthesis and benefit from essential resources like food, fiber, and timber, derived from the primary metabolites. Moreover, a diverse range of specialized metabolites, including terpenoids, phenylpropanoids, polyketides, and alkaloids, which, though not directly essential for plant growth and development, also play regulatory roles in plant growth and defense, blurring the biochemical boundaries between metabolite classes (Erb and Kliebenstein, 2020). Many plant specialized metabolites can serve as botanical medicines for humans (Pagare et al., 2015). Throughout centuries, people worldwide have sought remedies for common ailments from various parts of plants, such as flowers, stems, leaves, roots, fruits, and seeds (Petrovska, 2012). Although the active ingredients were not fully understood, ancient pharmacopoeias meticulously documented the precise applications and morphological features of numerous medicinal plants. Various plant specialized metabolites, including camptothecin (Kai et al., 2015), paclitaxel (Xiong et al., 2021), vinblastine (Kellner et al., 2015), and artemisinin (Shen et al., 2018), have been used to treat challenging diseases like cancer, malaria, and cardiovascular conditions, previously deemed incurable by ancient healers. Plant specialized metabolites are attracting considerable attention in the medical and health industries as eco-friendly, renewable, and sustainable natural products with promising potential for future innovation (Hussein and El-Anssary, 2019).

The plant kingdom encompasses diverse specialized metabolites, exceeding 200,000 types, exhibiting variations in function, distribution, and chemical structure (Kessler and Kalske, 2018). Analysts have extracted specialized metabolites from plant tissues and utilized a diverse array of advanced biochemical and sequencing technologies to analyze their structure and content (Abubakar and Haque, 2020; Ma and Qi, 2021; DiBello et al., 2023), leading to significant advancements in understanding plant specialized metabolites. However, the low concentrations of specialized metabolites in plant tissues render isolating substantial quantities of these compounds challenging, limiting their widespread adoption (Pagare et al., 2015). As a result, there are efforts to enhance the production of specific plant specialized metabolites through metabolic engineering and synthetic biology, aiming to establish large-scale, efficient, and eco-friendly manufacturing processes (Liu and Stewart, 2015). Qu et al. (2015) identified and functionally characterized key genes in the seven-step biosynthesis pathway from tabersonine to vindoline in *Catharanthus roseus*. They reconstituted this pathway in yeast and demonstrated its ability to convert exogenously supplied tabersonine into vindoline, highlighting the potential for metabolic engineering to enable scalable production of this important precursor to the anticancer drug vinblastine. Moreover, CRISPR/Cas-mediated precision genome editing technology holds promise for altering the production of specific plant specialized metabolites in medicinal plants (Mipeshwaree Devi et al., 2023). The effective application of these cutting-edge technologies provides a valuable blueprint for the large-scale production of diverse plant specialized metabolites. However, these endeavors require a deep understanding of the biosynthesis pathway of the desired specialized metabolites. The shikimate, methylerythritol phosphate, mevalonic acid, and tricarboxylic acid cycle pathways, along with other central metabolic pathways, provide essential precursors and building blocks for the biosynthesis of specialized metabolites. These fundamental pathways are highly conserved among plant species (Jamwal et al., 2018). Nonetheless, the later stages of plant specialized metabolite biosynthesis pathways are notably complex, with considerable diversity across cell types, developmental stages, and environmental cues (Li et al., 2020). This complexity involves a wide array of genes, transcription factors, signaling pathways, and enzymatic reactions, which vary significantly among plant species, thus making understanding these pathways challenging. For instance, recent advances in identifying missing steps and critical enzymes in the vinblastine biosynthesis pathway highlight this complexity. Tatsis et al. (2017) identified and functionally characterized geissoschizine synthase and geissoschizine oxidase, which catalyse key transformations in the formation of the *Strychnos* alkaloid scaffold. Qu et al. (2018) discovered Redox1, Redox2, and stemmadenine-*O*-acetyltransferase, which mediate subsequent biosynthetic steps, including oxidation, reduction, and acetylation reactions. Caputi et al. (2018) completed the pathway by identifying the final missing enzymes, including precondylocarpine acetate synthase, dihydroprecondylocarpine acetate synthase, catharanthine synthase, and tabersonine synthase, which enable the formation of vinblastine precursors. These discoveries collectively highlight the complex enzymatic interplay required to generate chemical diversity in plants, as demonstrated by the biosynthesis of diverse alkaloid scaffolds from central intermediates.

Accurate assembly and comprehensive annotation of the nuclear genome are essential for facilitating the investigation of the key biosynthesis pathways of specialized metabolites in medicinal plants. The release of the genomic sequence of *Arabidopsis thaliana*, a flowering and model plant, marked the advent of the genomic era in plant research in 2000 (The Arabidopsis Genome Initiative, 2000). In the past two decades, more than a thousand plant genomes have been released, with many belonging to medicinal plants, as indicated by a noticeable increase in newly sequenced genomes of medicinal plants in the last five years (Cheng et al., 2021). Large, multi-agency genome sequencing projects, including the 10KP (10,000 Plant Genomes; Cheng et al., 2018), the 1K Medicinal Plant Genome Project (Su et al., 2022), and the Earth Biogenome Project (Lewin et al., 2018), have greatly expanded the number of sequenced medicinal plant genomes. As of November 2024, 107 medicinal plant genomes are cataloged in the 1K Medicinal Plant Genome Database, with the total number of released medicinal plant genomes exceeding this number. Coupled with advanced assembly algorithms, the cutting-edge long-read, high-precision sequencing technologies hold promise for achieving exceptional accuracy and continuity in genome assembly of medicinal plants, including those with large genomes or high heterozygosity. Notable examples include *Lilium davidii* var. *unicolor* (38 Gb, 2.2% heterozygosity; Xu S. et al., 2024), *Allium fistulosum* (12 Gb, 0.6% heterozygosity; Liao et al., 2022), *Cannabis sativa* (800 Mb, ~2.0% heterozygosity; Gao et al., 2020; Wei et al., 2024), and *Crocus sativus* (7.6 Gb, 2.0% heterozygosity; Xu Z. et al., 2024). Through such high-quality plant genomes, diverse methodologies can be employed to discover candidate genes associated with plant specialized metabolite biosynthesis. Comparative genomics enables the exploration of the evolutionary mechanisms influencing diverse biosynthesis pathways across species. Functional genomics explores gene expression patterns and functions to identify candidate genes involved in plant specialized metabolite biosynthesis pathways. Moreover, the rapid development of various omics techniques such as transcriptomics, proteomics, and metabolomics greatly enhances the efficiency and accuracy of genome mining for plant specialized metabolite biosynthesis pathways.

This review aimed to provide a comprehensive overview of recent advances in medicinal plant genome sequencing and assembly, highlighting key findings that have significantly contributed to understanding specialized metabolite biosynthesis pathways. We discuss how combining various omics technologies has unraveled the complex interactions between genes, enzymes, and metabolites. Furthermore, we examine the recent advances in integrating multi-omics in studying specialized metabolite biosynthesis in medicinal plants. Finally, we address the ongoing challenges and provide future directions in the field, emphasizing the need for extensive collaboration and innovative methodologies to fully harness the potential of medicinal plants for improving human health and well-being.

## 2 Genomic foundations: decoding the biosynthetic blueprint of medicinal plants

### 2.1 Unlocking genomic complexity through nuclear genome sequencing and assembly

In 2010, the nuclear genome of castor bean (*Ricinus communis*) was sequenced through Sanger sequencing, making the first medicinal plant to be sequenced (Chan et al., 2010). From 2010 to 2015, additional medicinal plant genomes were reported, including *Brassica oleracea* (Liu et al., 2014) and *Catharanthus roseus* (Kellner et al., 2015). During this period, next-generation sequencing technologies like Illumina, Roche 454, and SOLiD advanced rapidly, offering high throughput and reduced costs compared to Sanger sequencing. Consequently, these techniques emerged as the favored methods for plant genome sequencing. Since 2016, next-generation sequencing technology has led to the release of numerous medicinal plant genomes, with Illumina favored for its effectiveness. However, the relatively short length of the next-generation sequencing reads poses a hurdle during the assembly of highly repetitive or heterozygous segments within plant genomes. This limitation could potentially result in the omission of vital genomic details (Kersey, 2019).

Third-generation sequencing technologies, such as PacBio and Oxford Nanopore, circumvent this hurdle by generating sequencing reads spanning several thousand to tens of thousands of base pairs. These read lengths surpass next-generation sequencing technologies, facilitating the interpretation of complex genomic regions and enhancing genome assembly completeness (Jiao and Schneeberger, 2017). Despite the extended read length, third-generation sequencing data exhibit high error rates, requiring correction with next-generation sequencing data (Bleidorn, 2016). This correction process further increases sequencing costs. Although third-generation sequencing technologies were available since 2010 (Munroe and Harris, 2010), their application in decoding medicinal plant genomes began after 2016. The increasing availability of third-generation sequencing platforms and the reduced sequencing costs have effectively overcome this obstacle, leading to increased reports of published medicinal plant genomes generated through hybrid assemblies of next-generation and third-generation sequencing data, especially from 2020. With third-generation sequencing technology, genome assemblies of medicinal plants have achieved remarkable continuity, precision, and fewer assembly errors like misplacement and redundancy. Furthermore, techniques like optical mapping and high-throughput chromosome conformation capture aid in determining chromosomal positioning in genomes assembled from third-generation sequencing data. As an illustration, Cheng et al. (2021) employed high-coverage Nanopore sequencing alongside high-throughput chromosome conformation capture data to reconstruct the *Taxus wallichiana* genome, yielding a chromosome-level assembly of 10.9 Gb. This high-quality assembly enables further functional analysis of two isoenzymes involved in the paclitaxel biosynthesis pathway. Third-generation sequencing technology has also been utilized to enhance the assembly quality of the earlier versions of medicinal plant genomes. In general, sequencing and assembling of medicinal plant genomes is progressing towards decreased costs, improved accuracy, and higher continuity.

A telomere-to-telomere reference genome of a medicinal plant provides comprehensive genomic information, serving as a valuable blueprint for further exploration. There has been ongoing pursuit of telomere-to-telomere level genome assembly in recent years. This initiative seeks to create comprehensive reference genomes encompassing the entire length of chromosomes from one telomere to the other (Mc Cartney et al., 2022). Recent advancements in third-generation sequencing technology, notably the successful integration of Oxford Nanopore ultra-long sequencing with PacBio HiFi sequencing, have significantly eased the challenging process of assembling centromeric and other highly repetitive genomic regions. Recently, telomere-to-telomere level assemblies have been completed for several medicinal plants, including *Scutellaria baicalensis* (Pei et al., 2023), *Rhodomyrtus tomentosa* (Li F. et al., 2023), *Mentha suaveolens* (Yang et al., 2024a), *Isodon rubescens* (Yang et al., 2024b), *Peucedanum praeruptorum* (Bai et al., 2024), and *Rheum officinale* (Zhang et al., 2024), paving the way for accelerated and more comprehensive functional analyses.

**Figure 1:**
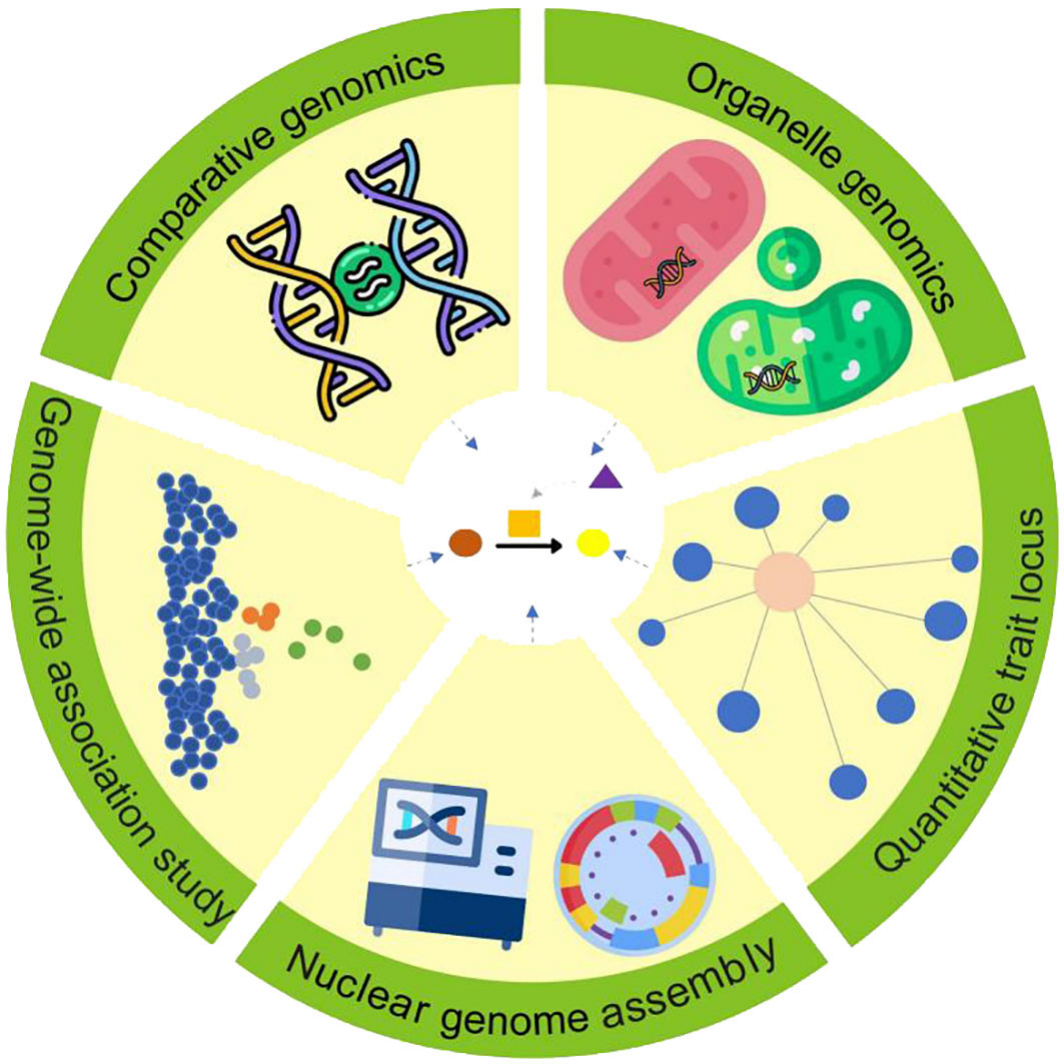
Multiple genomic approaches for uncovering specialized metabolic pathways in medicinal plants.

Despite notable advances in nuclear genome sequencing and assembly, fully elucidating specialized metabolite biosynthesis pathways in medicinal plants still demands the integration of diverse genomic techniques (**Figure 1**). Here, we focus on the renowned medicinal plant hemp (*Cannabis sativa* L.) as a prime example. Soorni et al. (2017) pioneered the use of genotyping-by-sequencing in *C. sativa* to reveal distinct genetic clusters within Iranian germplasm, identifying key genetic markers linked to specialized metabolite traits and providing a basis for functional studies. Building on this, Dehnavi et al. (2024) expanded genotyping-by-sequencing applications to identify novel loci related to flowering time, sex, and chemotyping in Iranian populations, demonstrating the value of population-level data in understanding trait variation. Enhancing genomic resources, Gao et al. (2020); Braich et al. (2020), and Wei et al. (2024) developed high-quality reference genomes for wild, medicinal, and seed *C. sativa*, respectively, providing essential templates for analyzing genetic diversity, exploring regulatory elements, and illuminating pathways in lipid and specialized metabolite biosynthesis. Ren et al. (2021) further extended these insights through large-scale whole-genome resequencing, reconstructing *C. sativa*’s domestication history and uncovering genetic divergences that shape distinct metabolic profiles in hemp and drug-type cannabis. Collectively, these advanced genomics methods create a comprehensive framework for deciphering regulatory networks governing specialized metabolite biosynthesis. Furthermore, pan-genomic approaches in medicinal plants, though still emerging, hold immense promise for uncovering genetic diversity across species and populations, identifying core and accessory genes involved in bioactive compound production, and discovering novel pathways linked to therapeutic properties (Zhou and Liu, 2022). As sequencing technology becomes more accessible and costs decline, combining high-quality medicinal plant genomes with other genomic data will increasingly facilitate a thorough understanding of specialized metabolite biosynthesis pathways in medicinal plants.

While current advancements in sequencing technologies have enabled the assembly of many medicinal plant genomes, there remains a significant demand to decode extremely complex genomes, such as those of autopolyploids and plants with very high heterozygosity, which present substantial challenges for accurate assembly. These complex genomes cannot yet be adequately resolved with existing methods, underscoring the need for the development of more sophisticated genomic sequencing technologies and novel algorithms specifically tailored to assemble such challenging genomes.

### 2.2 Deciphering biosynthetic gene clusters: pathways to metabolite diversity

In plant genomes, genes responsible for specialized metabolite biosynthesis are frequently organized into clusters termed BGCs, which represent specialized genomic regions containing groups of genes synthesizing specific metabolites or related groups of metabolites (Medema et al., 2015). These clusters contain non-homologous genes encoding enzymes for specialized metabolite synthesis, modification, and transportation (Nützmann et al., 2016). BGCs responsible for specialized metabolite biosynthesis in plant genomes vary in size from approximately 35 kilobases to several hundred kilobases, typically containing 3-10 closely spaced genes with similar expression patterns (Nützmann et al., 2016; Bharadwaj et al., 2021).

An exemplary BGC is the glucosinolate biosynthesis cluster in *Arabidopsis thaliana*, which synthesizes compounds for defense against herbivores and pathogens (Kroymann et al., 2003). Another well-known BGC, CYP76M7, produces diterpene phytoalexins in rice, crucial for defense against microbial infections (Swaminathan et al., 2009). Recent studies have identified additional BGCs of significant importance. For instance, *Schizonepeta tenuifolia* was found to have a bipartite BGC that controls the biosynthesis of *p*-menthane monoterpenoids through an inverted duplication event (Liu et al., 2023). In addition, a BGC encoding the biosynthesis of ferruginol, a diterpenoid conserved among plants in the Lamiaceae family, has been characterized in *Salvia officinalis* (Li H. et al., 2023). Furthermore, their study revealed that the biosynthesis of clerodane diterpenoids in the distinct lineages of the *Salvia* and *Scutellaria* genera exemplifies repeated evolution within Lamiaceae. Another notable BGC involves the polyketide precursor phloroisobutyrophenone of hyperforin, organized in a BGC in *Hypericum perforatum*, representing the first example of aromatic polyketide biosynthesis discovered in plants (Wu et al., 2022). Their study also revealed that the two hyperforin BGCs in *H. perforatum* originated independently through convergent evolution. In addition, several recent studies in rice and tomato have identified novel BGCs that enhance plant resilience and productivity. A six-gene fatty acid metabolic cluster (*FGC3*) in rice, conserved across the Poaceae family, regulates the synthesis of hydroxy fatty acids crucial for reproductive development and yield (Yang C. et al., 2024). In tomato, two BGCs that enhance phenolamide accumulation and drought resistance were identified; however, these clusters, along with the regulatory gene *SlMYB13*, were subjected to negative selection during domestication (Cao et al., 2024). Additionally, tomato possesses an acylsugar BGC that regulates the accumulation of insecticidal acylsugars, enhancing plant resilience against herbivores, with evolutionary evidence showing that this BGC co-localizes with steroidal alkaloid genes in Solanaceae (Fan et al., 2020). These findings underscore the significant potential of BGCs for advancing plant improvement. However, unraveling the evolutionary origins of BGCs is a complex and challenging endeavor. The existing reports suggest that BGCs emerge through gene duplication events, followed by the neofunctionalization of genes in the primary metabolic pathways (Bharadwaj et al., 2021). This is further supported by Wang et al. (2022), who demonstrated that gene duplication followed by divergence led to new enzymatic activities in the indole alkaloid biosynthetic pathway. Similarly, Kerwin et al. (2024) demonstrated that gene duplication within an acylsugar BGC in tomato led to the emergence of paralogs, including *SlASAT1-L*, which underwent regulatory divergence and acquired root-specific expression, enabling the evolution of a distinct acylsugar biosynthesis pathway separate from the trichome pathway.

**Figure 2:**
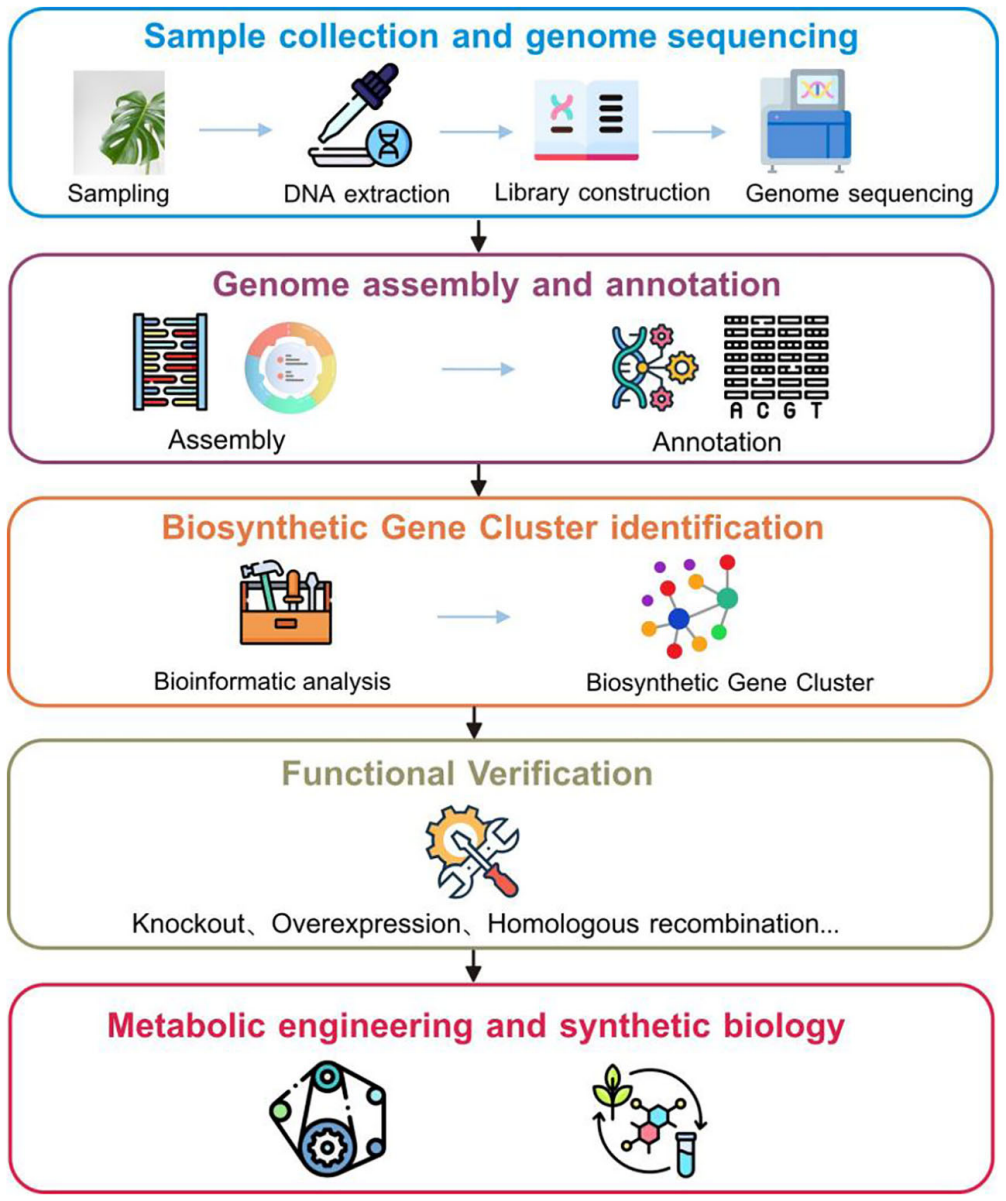
Genome-based workflow for identifying and engineering biosynthetic gene clusters in medicinal plants.

Identifying BGCs throughout the entire genome of medicinal plants could aid in understanding the pathways associated with plant specialized metabolite biosynthesis (**Figure 2**). Precise detection of BGC regions and their related functional genes in medicinal plants requires high-quality genome assembly and thorough annotation. This process enables the exploration and comprehension of pathways by highlighting enzymatic and non-enzymatic elements like transporters and regulators (Polturak and Osbourn, 2021). For example, Li et al. (2021) constructed a chromosomal-level genome assembly of *Avena strigosa*, elucidating the 12-gene avenacin BGC and clarifying the final two steps of avenacin synthesis. Li et al. (2022) constructed a high-fidelity genome assembly for sage (*Salvia officinalis*), and subsequent genomic analysis revealed a BGC region containing two pairs of diterpene synthase genes and several cytochrome P450 genes. The genes were within and adjacent to the cluster, coordinating distinct expression cascades that regulate diterpenoid production in the shoots and roots.

It is noteworthy that, unlike many microbial BGCs, plant BGCs generally do not contain all the genes necessary for the complete biosynthesis of a specialized metabolite. Instead, they often encode only a subset of the pathway, with additional biosynthetic components dispersed elsewhere in the genome (Nützmann et al., 2016). This fragmented arrangement complicates efforts to fully decipher the biosynthesis pathways for specialized metabolites in plants. Overcoming these challenges requires integrating data from BGCs with comprehensive genome-wide analysis, enabling the identification of missing pathway components and a deeper understanding of their regulatory interactions.

Future research on BGCs in medicinal plants could use synthetic biology to reconstruct BGCs in microbial hosts, enabling controlled mass production of medicinal compounds without the constraints of plant growth or environmental conditions. Artificial intelligence and machine learning models could be employed to discover novel BGCs from large genomic datasets, predict their therapeutic applications, and accelerate the identification of novel compounds. Additionally, genome-editing technologies like CRISPR/Cas9 could enhance bioactivity and yield by modifying BGCs to boost production or generate novel compounds. Focusing on BGCs that synthesize multifunctional metabolites could also yield plants that provide both medicinal benefits and resilience to environmental stresses, offering integrated solutions to global challenges.

### 2.3 Insights from comparative genomics: tracing metabolic pathway evolution in medicinal plants

Specialised metabolite biosynthesis pathways show varying conservation and specificity levels across plant species (Verma and Shukla, 2015). The pathways responsible for synthesizing the widely distributed specialized metabolites, such as flavonoids, alkaloids, terpenoids, and phenolic compounds, play a fundamental role in plant physiology and ecological interactions. These pathways are highly conserved among various plant species (Moghe and Last, 2015). For instance, the flavonoid biosynthesis pathways, essential for ultraviolet protection, coloration, and defense, exhibit minimal variation across plant species (Winkel-Shirley, 2001; Grotewold, 2006). Additionally, chalcone synthase, chalcone isomerase, flavanone 3-hydroxylase, and other conserved enzymes are prevalent across the genomes of numerous plant species (Wen et al., 2020). Conversely, certain specialized metabolite biosynthesis pathways are specific to particular medicinal plant species, producing distinctive bioactive compounds. These species-specific pathways significantly enhance the metabolite diversity of these plants. For example, the Madagascar periwinkle (*Catharanthus roseus*) is notable for its unique synthesis of the anti-cancer alkaloids vincristine and vinblastine, despite the presence of similar precursors such as strictosidine and tryptamine in other plant species (Qu et al., 2019). Similarly, the autumn crocus (*Colchicum autumnale*) synthesizes colchicine, which is renowned for effectively treating ailments such as gout and familial Mediterranean fever (Akram et al., 2012).

**Figure 3:**
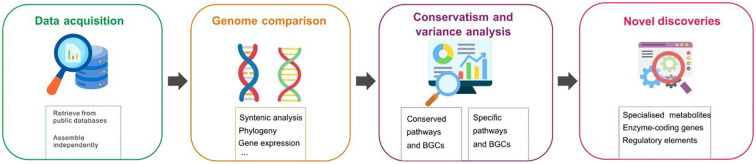
Comparative genomics for elucidating conserved and species-specific specialized metabolite biosynthesis pathways.

The emergence of species-specific pathways highlights the importance of comparative genomics to elucidate the origins and diversification of biosynthesis pathways (Bradbury et al., 2013). A comprehensive strategy integrating genomic data from diverse plant species is necessary for exploring specialized metabolite biosynthesis pathways in medicinal plants through comparative genomics (**Figure 3**). This is facilitated by the increasing accessibility of high-quality genomic data. Furthermore, analyzing synteny across plant genomes facilitates the identification of conserved regions and essential gene clusters involved in specialized metabolite biosynthesis. Syntenic analysis has successfully identified conserved gene clusters in indole alkaloid biosynthesis, as shown by Franke et al. (2019). Their study found that gene clusters in *Gelsemium sempervirens* are conserved with those in *Catharanthus roseus*, demonstrating how syntenic analysis can help discover genes in monoterpene indole alkaloid pathways, even in distantly related plants with different chemical profiles. Comparative genomics also explores the evolutionary relationships and divergence of specialized metabolite biosynthesis pathways among various species via reconstructing phylogenetic trees, examining the presence of polyploidy events, and analyzing expansions and contractions in gene families. These interspecies analysis enable the inference of the evolutionary trajectory of pathways and the identification of adaptation mechanisms specific to lineages. For instance, comparative genomic analysis of *Scutellaria baicalensis* and other Lamiaceae species revealed that its root-specific 4′-deoxyflavone biosynthesis pathway evolved through tandem and segmental gene duplications, coupled with subfunctionalization, enabling the recruitment and specialization of enzymes for flavonoid production (Zhao et al., 2019). Comparative genomics approaches have provided valuable insights into the origins and diversification of biosynthesis pathways of plant specialized metabolites. In a study on *Nepeta* species, Lichman et al. (2020) employed comparative genomics and ancestral enzyme reconstructions to reveal how iridoid biosynthesis, a key pathway for producing volatile metabolites like nepetalactones, was lost and subsequently re-evolved in the Nepetoideae subfamily of Lamiaceae. This work highlighted the role of gene duplication, enzymatic innovation, and metabolic gene clustering in driving the re-emergence of this pathway, illustrating the evolutionary flexibility and diversification of specialized metabolic processes in plants. In another study, the gene families involved in benzylisoquinoline alkaloid biosynthesis, including morphine and codeine, were examined in *Papaver somniferum* (Winzer et al., 2012). The study revealed a significant expansion in enzyme-coding gene families such as O-methyltransferases and cytochrome P450s, indicating an evolutionary advantage for diverse alkaloid production.

Comparative genomics analysis facilitates the discovery of new BGCs and pathways in medicinal plants, potentially uncovering lineage-specific pathways (Kautsar et al., 2017). Additionally, comparative genomics can also shed light on the regulatory mechanisms governing plant specialized metabolite biosynthesis in various ways (Li et al., 2020), including identifying promoter regions, enhancers, and other regulatory sequences. This enhances the understanding of gene expression changes in response to environmental stimuli and developmental signals. For example, Yin et al. (2023) conducted a comparative genomics analysis between *Lonicera macranthoides* and *L. japonica*, revealing sequence variations in the promoter regions of two collinear genes. These variations may explain differences in the enzymes and hederagenin-based saponins between the two species.

In summary, comparative genomics plays a vital role in unraveling specialized metabolite biosynthesis pathways in medicinal plant genomes by elucidating evolutionary origins, identifying new BGCs, and uncovering regulatory mechanisms governing the biosynthesis of specialized metabolites. Integrating genomic insights with ecological and evolutionary studies can help predict how climate change may impact the bioactive profiles of medicinal plants, thereby guiding conservation strategies to protect these valuable species and their unique chemotypes. Expanding comparative genomics beyond well-known medicinal families to underexplored plant lineages offers significant potential for discovering novel bioactive compounds. Such efforts can diversify the pharmacopeia, providing untapped therapeutic resources for modern medicine while enhancing the understanding of evolutionary adaptations in lesser-studied plants. This expansion is essential for broadening the range of medicinal compounds available for drug development and sustainable use.

### 2.4 Linking genetics with metabolites: genome-wide association studies and quantitative trait loci mapping in specialized metabolism

GWAS examines individual genomes from diverse plant populations, revealing genetic variations linked to plant specialized metabolite biosynthesis (Gupta et al., 2019). It captures natural genetic diversity arising from mutation, recombination, and selection, explaining specialized metabolite content variations. By examining extensive genomic datasets and phenotypic data, GWAS can reveal significant correlations between genetic markers and metabolite contents. This can identify the genomic regions containing essential candidate genes crucial for plant specialized metabolite biosynthesis. Moreover, GWAS can detect allele-specific effects through allele frequency and distribution among individuals with diverse metabolite phenotypes (Pastinen, 2010), offering insights into genetic variants impacting plant specialized metabolism. For example, Xiao et al. (2021) sequenced the nuclear genomes of 300 unrelated *Populus tomentosa* individuals and used GWAS to identify numerous candidate genes linked to salicylic acid biosynthesis through single nucleotide polymorphisms (SNPs). Kainer et al. (2019) utilized over two million SNPs acquired through whole-genome resequencing of 480 individuals of *Eucalyptus polybractea* to analyze the genetic framework governing terpene and biomass-related traits. The study identified a new set of candidate genes associated with terpene oil yield in *Eucalyptus* species. Sun et al. (2024) conducted GWAS on 180 *Dioscorea zingiberensis* accessions to identify genomic regions linked to diosgenin biosynthesis. They highlighted a significant SNP transversion within *CYP94D144*, a member of the P450 gene family, underscoring its crucial role in diosgenin production in tubers.

QTL mapping detects genomic regions linked to quantitative traits like specialized metabolite contents through genetic linkage maps (Kearsey and Farquhar, 1998), offering advantages in observing trait segregation and performing genetic mapping in controlled breeding populations. QTL mapping allows for the discovery of QTLs governing the biosynthesis of target specialized metabolites and containing gene clusters encoding enzymes, transporters, and regulatory factors. Therefore, this technique sheds light on the genetic framework and aids in uncovering essential genes and regulatory elements coordinating plant specialized metabolite biosynthesis pathways. For example, Yu et al. (2023) employed over 5,000 SNP markers to construct the first genetic map of *Epimedium*, uncovering 46 consistent QTLs linked to Epimedin C, total flavone content, and leaf size. Ma et al. (2018) utilized both simple sequence repeats and SNP markers to develop a genetic map for *Camellia sinensis*, unveiling 10 QTLs controlling caffeine, theobromine, their combination, and ratio in tea plants, thus identifying the QTL influencing caffeine content. Huang et al. (2024) investigated seed size and weight in castor bean using QTL analysis across four populations. Two novel QTL clusters were identified, and a total of 44 and 30 QTLs were detected for seed size and weight, respectively. Understanding QTL-environment interactions clarifies the influence of environmental factors on specialized metabolism, providing insight into how plant specialized metabolite accumulation responds to diverse environmental conditions (El-Soda et al., 2014).

GWAS scans the entire genome for SNPs in large populations, while QTL mapping focuses on controlled crosses. Despite methodological disparities, both are potent tools for elucidating the genetic mechanisms underlying the specialized metabolite biosynthesis pathways in medicinal plant genomes. Ongoing advancements in genome sequencing techniques refine the accuracy of association mapping and QTL localization while enhancing the precision, sensitivity, and interpretation of biosynthesis pathways. Future directions in GWAS and QTL mapping of medicinal plants emphasize integrating multi-omics approaches to understand specialized metabolite pathways comprehensively, enabling precise identification of genes involved in specialized metabolite biosynthesis. Exploring wild relatives uncovers novel alleles that enhance specialized metabolism production, providing valuable genetic diversity that can be introgressed into cultivated medicinal varieties. Additionally, population-wide studies across diverse regions can reveal adaptive genetic variations influencing specialized metabolism, allowing the development of medicinal plants optimized for specific environments to maximize bioactive compound yield and medicinal efficacy.

### 2.5 Beyond the nucleus: exploring organelle contributions to specialized metabolism

In addition to the nuclear genome, organelle genomes, including chloroplast and mitochondrial genomes, are essential contributors to the biosynthesis pathways of plant specialized metabolites (Mackenzie and McIntosh, 1999; Zhang et al., 2020). The chloroplast genome contains genes crucial for photosynthesis and diverse metabolic pathways, influencing specialized metabolite synthesis, such as alkaloids, flavonoids, and terpenoids by synthesizing essential precursors like isopentenyl diphosphate and dimethylallyl diphosphate (Wang et al., 2019). In addition to the genes responsible for synthesizing these precursors, the chloroplast genome encodes enzymes involved in the subsequent stages of specialized metabolite production (Zhang et al., 2020). Chloroplasts are well-known for their role in carotenoid biosynthesis and housing genes encoding the enzymes pivotal for the methylerythritol phosphate pathway, isoprenoid precursor synthesis, and subsequent steps in carotenoid biosynthesis, such as phytoene synthase and phytoene desaturase, commonly present in the chloroplast genome (Sandmann, 2021). Primarily recognized for their role in energy production through oxidative phosphorylation, mitochondria harbor genes essential for plant specialized metabolite biosynthesis. Mitochondrial genomes encode enzymes that catalyze diverse metabolic pathways, including those pivotal to the production of amino acids, hormones, and coenzymes acting as precursors or cofactors for synthesizing numerous specialized metabolites (Møller et al., 2021). For example, Zeng et al. (2024) demonstrated that a mitochondrion-localized BAHD acyltransferase found in *Atropa belladonna*, particularly the 3β-Tigloyloxytropane Synthase, plays a crucial role in the biosynthesis of calystegine.

Furthermore, organelle genomes interact with the nuclear genome to regulate gene expression during specialized metabolism, establishing a complex regulatory network of metabolic pathways (Woodson and Chory, 2008). Retrograde signals from chloroplasts and mitochondria modulate nuclear gene expression, regulating specialized metabolite biosynthesis for bioactive compound production in medicinal plants (Crawford et al., 2018). Additionally, the structural organization of organelle genomes, mediated by evolutionary processes and environmental stressors, can impact the specialized metabolite contents by altering the expression of relevant genes or biosynthesis pathways (Xu et al., 2015; Gualberto and Newton, 2017).

In summary, comprehending the relationship between organelle genomes and specialized metabolite biosynthesis is crucial for elucidating the pathways responsible for specialized metabolite production in medicinal plants. Despite the simplicity of assembling organelle genomes compared to nuclear genomes, the role of organelle genomes in specialized metabolite biosynthesis remains largely unexplored in medicinal plants, presenting extensive research opportunities in organelle genomics. Future research in organelle genomics could focus on integrating plastid and mitochondrial data to reveal their cooperative roles in specialized metabolite regulation. Advances in genome editing, such as CRISPR/Cas9, offer opportunities to enhance specialized metabolite production by engineering organelle genomes, optimizing biosynthesis pathways. Comparative genomics across different medicinal species could also uncover unique adaptations in organelle genes that impact specialized metabolite biosynthesis, guiding breeding or engineering efforts for improved therapeutic properties. Additionally, creating “mutator” lines for controlled mutation of organelle genomes could help identify novel regulatory elements or biosynthetic genes, unveiling new links between organelle functions and specialized metabolism for targeted metabolic engineering.

## 3 Mapping gene expression for metabolite biosynthesis: insights from transcriptomics

Transcriptomics analyzes RNA transcripts by exploring their types, quantities, and functions under specific conditions. Plant transcriptomics is crucial in uncovering how plants regulate gene expression in response to environmental changes and internal developmental signals. Microarrays, quantitative PCR, and RNA sequencing are the common techniques used in plant transcriptomics. Microarrays enable simultaneous analysis of gene expression by hybridizing labeled RNA to DNA fragments. Quantitative PCR measures gene expression levels by amplifying RNA with specific primers and fluorescent probes. RNA sequencing entails high-throughput sequencing of RNA molecules in a sample, enabling comprehensive analysis of transcriptomes and identification of gene expression patterns. Among these methods, RNA sequencing offers a more comprehensive and less biased view of the transcriptome, making it the preferred choice in plant transcriptomics (Wang et al., 2009). Recent advancements in transcriptomic sequencing have improved accessibility and provided insights into gene expression dynamics. These advancements are particularly beneficial for studying specialized metabolite biosynthesis pathways of medicinal plants.

Differential expression analysis and gene co-expression network analysis are two widely used approaches that leverage gene expression levels to elucidate the genomic framework underlying plant specialized metabolite biosynthesis. By comparing gene expression levels across different conditions, differential expression analysis identifies genes regulated in response to factors influencing specialized metabolite biosynthesis (Sanchita and Sharma, 2018). This identifies differentially expressed genes encoding enzymes catalyzing crucial steps in specialized metabolite biosynthesis. For instance, differential expression analysis can identify the activated genes crucial for alkaloid biosynthesis when a plant increases the production of a specific alkaloid in response to a microbial infection (Singh et al., 2020). Importantly, differential expression analysis can reveal regulatory networks by identifying co-regulated genes under specific conditions, suggesting potential regulatory candidates controlling specialized metabolite biosynthesis. Moreover, differential expression analysis explores biosynthesis pathways by correlating differentially expressed genes with known pathways, aiding in identifying high-confidence genes for further investigation. Gene co-expression network analysis identifies clusters of functionally linked genes by analyzing gene expression correlations across samples (Aoki et al., 2007). The technique offers a systemic view that provides insight into the coordinated regulation of specialized metabolism and identifies crucial genes and regulatory hubs mediating the synthesis of key specialized metabolites (Zhang and Horvath, 2005). A key application of gene co-expression network analysis involves identifying co-expressed gene modules and aligning them with known metabolic pathways and gene regulatory networks. This process identifies connections between genes involved in plant specialized metabolite biosynthesis and reveals novel interactions and regulatory relationships that might not be evident in individual gene expression studies. gene co-expression network analysis identifies key regulatory genes and hubs that control specialized metabolite biosynthesis pathways, often acting as master regulators governing the expression of downstream genes involved in the synthesis and regulation of specialized metabolites. Additionally, gene co-expression network analysis allows for comparisons across various plant species, uncovering shared or divergent regulatory mechanisms mediating specialized metabolite biosynthesis through the construction and comparison of co-expression networks (Ovens et al., 2021).

Single-cell RNA sequencing is a revolutionary transcriptomics technique that reveals specialized metabolite biosynthesis pathways specific to cell types or developmental stages (Aldridge and Teichmann, 2020). It enables the determination of gene expression patterns at the cellular level, offering unprecedented resolution that overcomes the limitations of conventional transcriptomic methods. Importantly, single-cell RNA sequencing tracks developmental transitions and identifies regulatory elements by revealing gene expression variations between cells. For example, Sun et al. (2023) mapped the spatial organization of monoterpenoid indole alkaloids metabolism in *Catharanthus roseus* leaves using single-cell RNA sequencing, generating the biosynthesis model with localized transcripts of 20 MIA genes. The study categorized the monoterpenoid indole alkaloid pathway into three distinct cell types, and identified key transporters, offering insights for enhancing monoterpenoid indole alkaloid yields in plants through metabolic engineering and synthetic biology. In another study on *C. roseus* by Li C. et al. (2023), single-cell transcriptomics revealed the segregation of the leaf monoterpenoid indole alkaloid biosynthetic pathway across different cell types, identifying a reductase responsible for anhydrovinblastine production. The study highlighted cell-type-specific gene expression patterns within the root monoterpenoid indole alkaloid pathway. Wu et al. (2024) highlighted the significance of single-cell RNA sequencing in their study of *Hypericum perforatum*, where they discovered specialized “Hyper cells” responsible for hyperforin biosynthesis in leaves and flowers. This innovative approach allowed them to pinpoint the precise cellular context of the biosynthesis pathway, leading to the identification of four key prenyltransferases. As the cost of single-cell RNA sequencing continues to decrease, many single-cell transcriptome studies are anticipated to replicate these successful applications in medicinal plants to gain a deeper understanding of plant specialized metabolite biosynthesis pathways.

Another powerful tool, spatial transcriptomics, has revolutionized the understanding of plant specialized metabolite biosynthesis pathways by providing precise spatial details on gene expression patterns within plant tissues (Ståhl et al., 2016). This method enables precise gene expression mapping, identifying specific cell types or tissues with active genes linked to specialized metabolite pathways, thereby overcoming the limitations of single-cell RNA sequencing and conventional transcriptomics. Spatial transcriptomics uncovers unknown enzymes, regulators, and transporters essential for specialized metabolite biosynthesis, clarifying spatial regulation and revealing metabolic centers (Yin et al., 2023). This aids understanding specialized metabolite synthesis, accumulation, and cell interactions within regulatory networks. Spatial transcriptomics also facilitates the visualization of metabolic changes within intact plant tissues, offering insights into the temporal regulation and adaptation to environmental stimuli in plants. Successful applications of spatial transcriptomics have been reported in various plants, such as *Trillium govanianum* (Singh et al., 2017), *Selaginella moellendorffii* (Yang et al., 2023), *Solanum lycopersicum* (Song et al., 2023), and *Angelica glauca* (Devi et al., 2022). However, this technique is still relatively new, and its widespread adoption in medicinal plants may take time as researchers familiarize themselves with its applications and benefits.

Leveraging transcriptomics, future studies could focus on guiding metabolic engineering to enhance specialized metabolite production via CRISPR-based editing of key regulatory genes. Additionally, dissecting regulatory mechanisms, such as transcription factors and non-coding RNAs, will enable fine-tuning of biosynthetic pathways for improved yields. Environmental stress responses also present an important avenue; transcriptomics can elucidate how factors like drought or temperature influence metabolite pathways, leading to optimized cultivation strategies that enhance medicinal compound production under diverse conditions, ultimately improving sustainability.

## 4 Proteomic insights: uncovering functional molecules in metabolite pathways

As direct products of gene expression, proteins are the functional molecules that regulate various biochemical pathways, including those involved in plant specialized metabolite biosynthesis. The mechanisms and regulatory networks underlying specialized metabolite biosynthesis can be determined by examining the proteome—the entire complement of proteins expressed by a genome, cell, tissue, or organism at a particular time. Proteomics, the large-scale study of proteins, is a powerful approach to exploring specialized metabolism in plants (Chen and Harmon, 2006). Major proteomic techniques include mass spectrometry for protein identification and quantification, two-dimensional gel electrophoresis for protein separation, protein microarrays for high-throughput protein analysis, and liquid chromatography-mass spectrometry for complex protein mixture analysis (Aslam et al., 2016).

Advancements in these proteomic techniques have significantly enhanced studies of complex protein networks in medicinal plants. Proteomics is used to identify and characterize enzymes directly involved in specialized metabolite biosynthesis. For example, Qiu et al. (2021) utilized tandem mass tag quantitative labeling technology for the proteomic analysis of fulvic acid-treated tea leaves from the target plants and compared these findings with previous transcriptomic analysis results. The study identified several genes encoding key enzymes catalyzing starch and sucrose metabolism, phenylpropanoid biosynthesis, and triterpenoid biosynthesis. Protein expression changes can reveal regulatory proteins, such as transcription factors and protein kinases, that control specialized metabolite pathways (Li et al., 2020). For example, Ashokhan et al. (2024) utilized two-dimensional gel electrophoresis techniques to identify key proteins and enzymes involved in the response of *Azadirachta indica* to abiotic stresses. Their analysis revealed differential regulation of proteins associated with critical biological processes, highlighting the potential of proteomics for pinpointing key genes and enzymes that could enhance stress tolerance and bioactive compound production, such as azadirachtin. Furthermore, proteomic analysis of post-translational modification processes, such as phosphorylation, glycosylation, and ubiquitination, can reveal how these modifications influence enzyme function, stability, and interactions, providing deeper insights into specialized metabolism regulation (Dai Vu et al., 2018). For example, Kumari et al. (2021) generated an extensive proteomic reference map of *Picrorhiza kurroa*, identifying over 5,000 proteins across various organs and developmental stages. The study also highlighted the significant roles of post-translational modifications in *P. kurroa*’s adaptation to high-altitude environmental fluctuations.

Despite these advancements, the application of proteomics in plants still faces several challenges. First, the diversity and complexity of plant proteomes and the presence of specialized metabolites render protein extraction, identification, and quantification difficult (Chen et al., 2021). Second, there is a lack of functional annotation for many proteins, highlighting the need for integrative approaches combining proteomics, genomics, transcriptomics, and metabolomics (Subramanian et al., 2020). Third, there is a need to enhance the sensitivity, accuracy, and throughput of proteomics, especially for detecting low-abundance proteins and complex post-translational modifications (Smith and Rogowska-Wrzesinska, 2020).

In summary, proteomics offers invaluable insights into the functional dynamics of specialized metabolism in plants. Proteomics also enhances our understanding of plant biochemistry and opens new avenues for the biotechnological exploitation of medicinal plants by unraveling complex protein networks and regulatory mechanisms. Advancing proteomics for studying specialized metabolism in medicinal plants involves leveraging cutting-edge mass spectrometry technologies to enhance sensitivity and accuracy, particularly for low-abundance proteins. Proteogenomics can uncover novel enzymes involved in biosynthetic pathways, aiding in new discoveries. Integrating CRISPR/Cas9 with proteomics allows functional validation of specific proteins, accelerating enzyme characterization. Additionally, establishing standardized proteomic protocols is crucial for ensuring consistency and reproducibility, promoting broader adoption and reliability of proteomic data in medicinal plant research.

## 5 Metabolite profiles and pathways: a metabolomics perspective on medicinal plants

Metabolomics is a post-genomics tool for investigating small molecule metabolites to provide insights into metabolic states (Hollywood et al., 2006). Plant metabolomics captures the dynamic interactions among genetic, environmental, and physiological factors affecting metabolism. Metabolomics employs various analytical methods for metabolite analysis, including nuclear magnetic resonance spectroscopy, mass spectrometry, and various chromatographic techniques. Nuclear magnetic resonance spectroscopy uses magnetic characteristics of atomic nuclei to reveal molecular structures, facilitating analysis of plant specialized metabolites (Deborde et al., 2017). Mass spectrometry ionizes and separates metabolites based on their mass-to-charge ratio, enabling the identification and measurement of several plant specialized metabolites (Jorge et al., 2016). Chromatographic techniques, such as gas chromatography-mass spectrometry and liquid chromatography-mass spectrometry, separate and analyze mixture components through interactions with stationary and mobile phases, enabling the identification and quantification of individual compounds in complex samples (Patel et al., 2021). The metabolomics analytical techniques encompass several statistical and bioinformatics methodologies. These include multivariate analysis tools like principal component analysis and partial least squares-discriminant analysis, along with pathway analysis methods, statistical tests, and machine learning algorithms such as support vector machines, random forests, and neural networks (Cambiaghi et al., 2017). Incorporating these methodologies could aid thorough metabolomics analysis to unravel the biosynthesis pathways of plant specialized metabolites. For instance, Yang J. et al. (2024) introduced a novel approach, widely targeted metabolite modificomics, integrating ultra-high performance liquid chromatography coupled with quadrupole-linear ion trap and Exactive-Orbitrap mass spectrometry. This method facilitates precise detection of plant-modified metabolites through targeted mass transition identification. When applied to tomato research, it identified over 34,000 signals and annotated 2,118 metabolites, spanning 125 modification types, including disease-linked metabolites. This method shows strong potential for advancing comprehensive metabolic profiling, identifying biomarkers, and discovering novel bioactive compounds in medicinal plants.

One of the primary objectives of plant metabolomics is to identify specialized metabolites by detecting, measuring, and understanding the structures of both known and unknown compounds. This expands our understanding of the diversity of plant chemicals (Nakabayashi and Saito, 2013). Moreover, metabolomics explores the complex metabolic pathways within medicinal plants (Scossa et al., 2018). Metabolomics-driven strategies map specialized metabolite biosynthesis by determining enzymatic reactions, intermediary metabolites, and regulatory mechanisms to identify key enzymes. This understanding helps identify key metabolites and provides insights into their biosynthesis, regulation, and environmental interactions. Moreover, integrating plant metabolomics with genomic data enables the characterization of enzyme activities by assessing changes in metabolite concentrations pre- and post-reactions and evaluates specificity through control experiments and inhibition studies. Metabolomics is also a potent tool aiding gene discovery (Patel et al., 2021). It offers valuable insights into the regulatory networks of specialized metabolism by correlating metabolite abundance changes with corresponding alterations in gene expression. Furthermore, the advanced computational algorithms enable the identification of candidate genes encoding enzymes catalyzing specific biosynthesis pathways, facilitating targeted gene discovery. Through metabolite-gene association studies, metabolomics helps uncover the genetic factors controlling the synthesis of crucial specialized metabolites and reveals the complex interplay between various metabolic pathways and regulatory elements. In addition, metabolomics is employed in GWAS and QTL studies to reveal loci linked to metabolite variations, aiding gene discovery for specialized metabolite biosynthesis (Luo, 2015; Sharma et al., 2021).

Like transcriptomics, metabolomics has advanced to single-cell resolution and spatial analysis, enhancing our understanding of metabolic diversity at the microscopic level. Single-cell metabolomics provides precise metabolite characterization within cells, and reveals cell-specific metabolic profiles and heterogeneity across tissues (Zenobi, 2013). Integrated genomics and metabolomics can identify key biosynthetic genes and pathways, elucidating cellular regulatory mechanisms. Additionally, metabolomics reveals rare metabolites crucial for medicinal properties, offering insights into the dynamic nature of plant metabolism. Spatial metabolomics identifies specialized metabolite biosynthesis pathways by mapping metabolite distributions and visualizing their organization, revealing key areas and gradients (Alexandrov, 2023). The technique provides insight into spatial coordination of metabolic processes by integrating genomics to elucidate spatial regulation, gene expression, and metabolic interactions.

Future directions in metabolomics for studying specialized metabolism in medicinal plants include targeted identification of rare bioactive compounds, developing a comprehensive digital metabolome library for plant authentication and discovery, integrating traditional knowledge with scientific validation, and investigating the functional roles of specialized metabolites in plant defense and environmental interactions. These approaches aim to identify novel therapeutic compounds, provide scientific backing to ethnobotanical practices, and uncover the ecological significance of specialized metabolites, ultimately bridging traditional knowledge with modern biochemical insights.

## 6 Integrative multi-omics: a comprehensive view of biosynthesis pathways in medicinal plants

**Figure 4:**
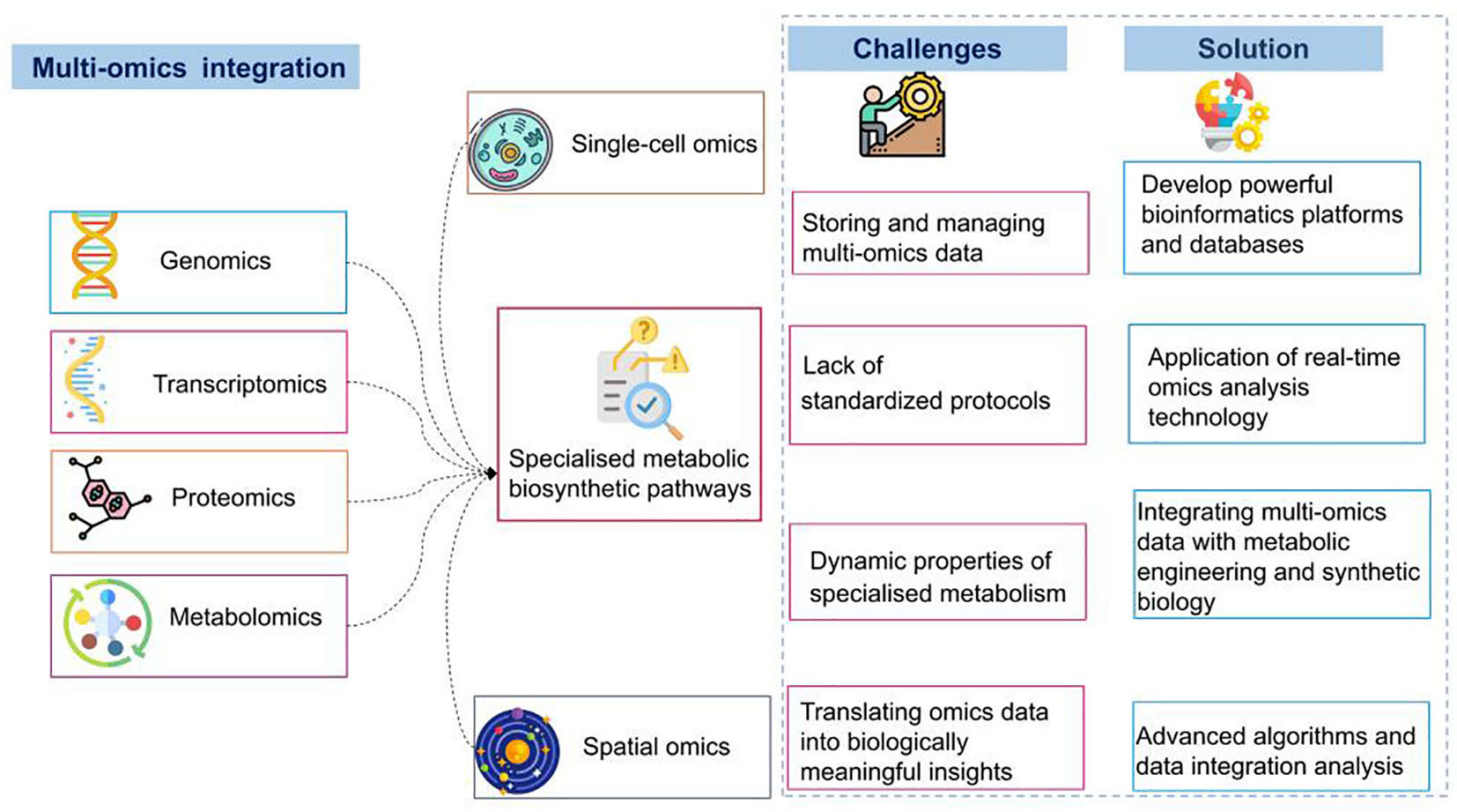
Overview of multi-omics integration for deciphering specialized metabolic pathways: challenges and solutions.

Integrating multi-omics data is an effective strategy for unraveling the complex specialized metabolite biosynthesis pathways in plants (Yang et al., 2023). This holistic approach uses the strengths of each omics technology (**Table 1**), to understand biosynthesis networks through various integration strategies (**Figure 4**). One such strategy involves correlation analysis, which links different omics data, such as gene expression and metabolite data, to identify co-regulated genes and metabolites and provide insights into their interactions. For example, Rubio-Rodríguez et al. (2021) found that methyl jasmonate enhances the production of total phenolic compounds in *Castilleja tenuiflora* without affecting growth. The study revealed a significant positive correlation between verbascoside and aucubin contents and the expression of key biosynthesis genes, thereby elucidating the molecular basis of specialized metabolite biosynthesis in this medicinal plant. Network-based approaches construct multi-layered networks integrating genomic, transcriptomic, proteomic, and metabolomic data, mapping interactions and regulatory pathways (Zhou et al., 2020). For instance, Ran et al. (2020) developed “Plant Regulomics”, an interface combining transcriptomic and epigenomic data, offering tools to identify regulatory factors and visualize interactions. This aids the functional characterization of genes, genomic loci, and associated regulatory mechanisms. Machine learning and computational modeling use algorithms to integrate omics data, forecast pathways, and identify regulatory nodes (Ma et al., 2014; Singh and Bharadvaja, 2021).

**Table 1:** Summary of major techniques, characteristics, usages, advantages, limitations, and future trends in genomics, transcriptomics, proteomics, metabolomics, and multi-omics integration for studying specialized metabolism in plants.

Omics Type	Major Techniques	Characteristics	Usages	Advantages	Limitations
Genomics	High-throughput sequencing	Reveals the complete DNA sequence	Genome assembly, gene identification, evolutionary studies	High accuracy, comprehensive genomic information, improves transcriptomic data quality, provides a reference for proteomics data	High cost, complex data analysis, requires extensive computational resources; polyploidy, heterozygosity, and unknown genome size or chromosome number increase costs and complexity
Transcriptomics	Microarrays, quantitative PCR, RNA sequencing	Analyzes RNA transcripts, gene expression levels	Gene expression profiling, studying gene regulation	Sensitive, high-throughput, comprehensive view of transcriptome	Potential biases, requires high-quality RNA samples, complex data analysis
Proteomics	Mass spectrometry, two-dimensional gel electrophoresis, protein microarrays, liquid chromatography-mass spectrometry	Identifies and quantifies proteins, examines protein-protein interactions	Characterizing enzymes, studying protein modifications, identifying regulatory proteins	Detailed protein information, identifies functional dynamics	Complex sample preparation, high cost, requires advanced instrumentation
Metabolomics	Nuclear magnetic resonance spectroscopy, mass spectrometry, chromatographic techniques	Profiles metabolites, explores metabolic pathways	Identifying and quantifying metabolites, mapping biosynthesis pathways	Comprehensive metabolic profiles, identifies key metabolites	Complex data analysis, requires advanced analytical techniques, high cost
Multi-Omics Integration	Correlation analysis, network-based approaches, machine learning and computational modeling	Integrates data from multiple omics layers to map interactions and regulatory pathways	Understanding complex biosynthesis networks, identifying co-regulated genes and metabolites	Holistic understanding, comprehensive analysis of interactions	Data integration challenges, requires advanced bioinformatics tools, high computational demands

Recently, there has been an increase in integrated multi-omics studies focusing on medicinal plants; however, integrating multi-omics data to uncover specialized metabolite pathways in medicinal plants faces challenges. First, managing extensive multi-omics data can be overwhelming, requiring computational resources and advanced bioinformatics tools for efficient analysis (Shahrajabian and Sun, 2023). Consequently, analysts must be proficient in both software and hardware domains. Second, the lack of standardized protocols for generating and integrating omics data introduces variability and reproducibility challenges, complicating the comparison and integration of datasets. Addressing this requires robust methodologies that employ advanced statistical and computational techniques to integrate the diverse omics layers. Third, the dynamic characteristics of specialized metabolism, influenced by developmental stages, environmental conditions, and stress responses, make data acquisition more complex (Li et al., 2020), necessitating time-series data and advanced modeling techniques. Furthermore, translating integrated omics data into biologically meaningful insights is complicated because identifying key regulatory nodes and understanding their roles in specialized metabolite biosynthesis pathways involves extensive validation experiments.

Deciphering these challenges could pave the way for future research and development opportunities. Developing robust bioinformatics platforms and databases that facilitate seamless data integration and analysis while supporting multi-omics data standardization and providing intuitive interfaces operable by researchers of varying expertise levels is crucial. Establishing standardized protocols for omics data would improve comparability and reproducibility, enabling reliable integration. Furthermore, implementing real-time omics analysis technologies, such as live-cell imaging combined with real-time transcriptomics and metabolomics, enables the capturing of dynamic changes in specialized metabolism and provides insights into the temporal regulation of metabolic pathways. Metabolic pathways can be predicted and regulatory nodes can be identified with higher accuracy using advanced algorithms for data integration and analysis. These algorithms, developed through machine learning and artificial intelligence, aid in handling the complexity and volume of multi-omics data. Integrating multi-omics data with metabolic engineering and synthetic biology through techniques such as CRISPR/Cas9 gene editing could enhance the production of valuable specialized metabolites in medicinal plants. Additionally, encouraging collaboration among researchers, institutions, and industries would expedite the progress of multi-omics integration, facilitating the sharing of data, tools, and expertise to overcome limitations and stimulate innovation. By addressing these challenges and adopting these propositions, we can enhance the understanding of specialized metabolite biosynthesis in medicinal plants based on multi-layer omics data.

## 7 Future directions and implications in metabolic research of medicinal plants

This review highlights significant advancements in understanding specialized metabolite biosynthesis pathways in medicinal plants through integrated multi-omics technologies. Comprehensive genome sequencing of medicinal plants, has unraveled complex biosynthesis pathways, revealing key BGCs and regulatory mechanisms. Advances in third-generation sequencing technologies, transcriptomics, proteomics, and metabolomics have provided insights into the genetic and biochemical networks underpinning specialized metabolite production. Notably, the identification and functional characterization of BGCs have paved the way for metabolic engineering and synthetic biology applications. Comparative genomics has elucidated the origins and diversification of specialized metabolite pathways, while GWAS and QTL mapping have identified genetic variations linked to metabolite production.

Integrated multi-omics presents a promising avenue for enhancing our understanding of interactions between genes, proteins, and metabolites, potentially unraveling novel specialized metabolites with therapeutic potential. Future research should focus on overcoming challenges related to data integration, standardization, and metabolism dynamics. Developing robust bioinformatics platforms and leveraging machine learning for predictive modeling would both be crucial in this endeavor. Moreover, advancing technologies such as single-cell and spatial omics would provide deeper insights into the cell-specific and spatial regulation of specialized metabolite biosynthesis. The application of CRISPR/Cas9 and other gene-editing tools could facilitate the metabolic engineering of medicinal plants to enhance specialized metabolite production. Collaboration among researchers, institutions, and industries is essential to drive innovation and translate findings into the development of sustainable natural products.
